# Are low-trauma fractures all fragility fractures? Insights into musculoskeletal and body composition characteristics of community-dwelling post-menopausal women with a recent fracture

**DOI:** 10.1007/s40520-025-03154-w

**Published:** 2025-08-13

**Authors:** Varvara Chatzipetrou, Thierry Chevalley, Ivan Padlina, Marina Portela, Serge Ferrari, Emmanuel Biver

**Affiliations:** Division of Bone Diseases, Department of Medicine, Geneva University Hospitals, Faculty of Medicine, University of Geneva, Rue Gabrielle-Perret-Gentil 4, 1205 Geneva, Switzerland

**Keywords:** Sarcopenia, Obesity, Fragility fracture, Osteoporosis, Bone microstructure

## Abstract

**Background and aims:**

The incidence of fragility fractures is increasing among community-dwelling postmenopausal women. Sarcopenia and obesity are significant risk factors for fractures, independent of osteoporosis. This study aims to investigate the prevalence of sarcopenia and obesity, as well as bone microstructure, according to osteoporotic status and fracture sites in older women with recent low-trauma fractures.

**Methods:**

This cross-sectional study included 135 community-dwelling postmenopausal women aged 65 and older, evaluated within six months of experiencing a low-trauma fracture (resulting from a fall from standing height or less) occurring at the humerus, proximal femur, vertebrae, pelvis, forearm, or ankle. Participants were recruited either prospectively through the Fracture Liaison Service (FLS) at the Bone Disease Department of Geneva University Hospitals (HUG) (*n* = 90) or retrospectively from the Geneva Retirees Cohort (GERICO) (*n* = 45). Bone mineral density (BMD) and body composition were assessed using dual-energy X-ray absorptiometry (DXA), muscle strength was measured by handgrip strength (HGS), and bone microstructure was evaluated using high-resolution peripheral quantitative computed tomography (HR-pQCT).

**Results and discussion:**

The prevalence of sarcopenia varied, with the overall prevalence across all definitions being 25%. It was significantly higher in osteoporotic women than in women with normal BMD (39% versus 6%, respectively, *p* = 0.014) and osteopenia (39% versus 20% respectively, *p* < 0.05). The prevalence of obesity was 24%, with particularly high rates observed among women with normal BMD (61%) and those with ankle or humerus fractures (43%, and 38%, respectively). Notably, 33% of women with major osteoporotic fractures (MOF) presented with normal BMD and without sarcopenia or obesity.

**Conclusions:**

A significant proportion of women with apparent low-trauma MOF does not have osteoporosis nor alterations of body composition (sarcopenia or obesity). The determinants of fracture risk in these women remain unclear and further investigations are required to better address secondary fracture prevention in this context.

**Supplementary Information:**

The online version contains supplementary material available at 10.1007/s40520-025-03154-w.

## Introduction

Falls and fractures represent a major public concern, as the population ages and the prevalence of falls varies across countries [1]. Among individuals aged 50–79 years old in 36 European countries, the age-standardized incidence of falls ranged between 3.0 and 52.5 falls/100 person years among women [2, 3]. The annual number of fragility fractures due to falls is projected to increase by over one million within a decade [4]. By 2040, the number of older adults at risk for osteoporotic fractures is expected to double [5].

The financial burden of fragility fractures is substantial. An over 50% increase in annual fracture incidence is expected to drive direct healthcare costs beyond $60 billion by the end of 2025 [6–8]. In 2019 alone, osteoporotic fractures accounted for approximately 4.5% of total healthcare expenditure in Switzerland (€3.4 billion out of €74.9 billion), a proportion significantly higher than the EU27 + 2 (European Union plus the United Kingdom and Switzerland) average of 3.5% [9]. Among women aged 50 and 80 years old, the lifetime risk of experiencing a major osteoporotic fracture (MOF, defined as low-trauma (LT) fractures of the hip, vertebrae, humerus, forearm) is 46.4% and 31.7%, respectively [10].

Therefore, there is an urgent need to identify community-dwelling individuals at risk of falls and fractures so that they may benefit from primary prevention programs. Osteoporosis remains a major risk factor for fractures [11]. Although LT fractures, defined as a fracture resulting from a fall from standing height or less occurring at the humerus, proximal femur, vertebrae, pelvis, forearm, or ankle are considered fragility fractures [12], about half of these fractures are not associated with low BMD and occur in women without osteoporosis [4, 12–16]. According to the Rotterdam and SOF studies respectively, 55.9% and 63.2% of post-menopausal women who sustained LT fractures were not osteoporotic [16, 17].

Sarcopenia is a geriatric syndrome characterized by the progressive loss of muscle mass and function, and it is recognized as a key, potentially modifiable risk factor for falls and fractures [3]. Individuals with sarcopenia have a significantly higher risk of fractures compared to their non-sarcopenic counterparts [3, 18, 19]. Among older adults, the reported prevalence of sarcopenia ranges from 0.3 to 73.0%, depending on the definition used and the population studied [19]. While multiple definitions of sarcopenia have shown associations with osteoporosis and fracture risk, their predictive value for fractures often diminishes after adjustment for femoral neck BMD [20, 21]. Nevertheless, sarcopenia remains strongly associated with MOF, particularly hip fractures [22]. In addition, osteosarcopenia, the coexistence of osteoporosis and sarcopenia, is an increasingly recognized condition that confers a higher risk of LT fractures compared to either condition alone [23].

Obesity, in contrast, is traditionally considered protective against hip and pelvic fractures due to increased BMD and greater adipose tissue in obese individuals which may reduce the mechanical impact of falls [24, 25]. Nevertheless, obesity is also associated with elevated risk fracture, especially in the upper limbs and ankles, likely because of the chronic inflammation associated with obesity and higher forces exerted during falls [26–28].

In this study, we aimed to investigate musculoskeletal and body composition characteristics in community-dwelling, postmenopausal women with a recent low-trauma fracture. To date, the associations between sarcopenia, obesity, and bone microstructural defects with fracture risk have primarily been explored in cohort studies comparing individuals with and without prevalent or incident fractures. However, the actual prevalence of these musculoskeletal conditions among newly fractured women, stratified by osteoporotic status and fracture site, remains unclear. We hypothesized that sarcopenia and/or obesity may be contributors of “fragility” fractures in non-osteoporotic women. We also investigated the proportion of women with low-trauma fracture, without “fragility” characteristics regarding BMD and bone microstructure, sarcopenia and obesity.

## Materials and methods

### Subjects

The present analysis is an observational descriptive study of 135 post-menopausal women over the age of 65 years, evaluated within the first six months following a low-trauma fracture, defined as a fracture resulting from a fall from standing height or less occurring, at the humerus, proximal femur, vertebrae, pelvis, forearm, or ankle. Participants were recruited consecutively either prospectively through the FLS of the bone disease department at HUG (*n* = 90) or retrospectively from participant visits of the GERICO, a cohort study investigating the predictive factors of fracture (*n* = 45) [29]. The nature of the study was descriptive.

Participants were community-dwelling women without severe comorbidities or disabilities (Charlson Comorbidity Index (CCI) ≥ 4 points, with an estimated 10-year survival rate of ≥ 50%) [30, 31]. The inclusion criteria were the following: age > 65 years, a LT fracture at bone sites including humerus, proximal femur, vertebrae, forearm (all considered as MOF), or pelvis and ankle regardless of the therapeutic intervention (surgical or conservative treatment). Women with severe comorbidities, especially active cancer and end-stage renal disease were excluded because of the higher prevalence of pathological fractures in these patients (metastatic bone disease and renal osteodystrophy related to chronic kidney mineral bone disorder, respectively). Women with moderate to severe cognitive impairment, documented either through medical records (clinical dementia rating ≥ 2 or mini-mental state examination score < 18) [32, 33] or clinically diagnosed based on an inability to follow simple orders), were also excluded due to potential difficulties in understanding and participating in the assessments. Written informed consent was obtained for all participants, and the study protocol was approved by the Geneva University Hospitals’ Research Ethics Committee.

All participants underwent a medical visit 4 to 6 months following the index fracture. During this visit, they completed questionnaires regarding demographic characteristics and behavioral factors. Detailed medical histories were recorded. Information on both current and prior fractures, as well as history of falls, was also collected. The CCI was calculated for each participant [30]. The FRAX tool (femoral neck BMD included in the algorithm) without the index fracture was used to estimate 10-year probabilities of MOF using country-specific data [34]. The objective was to assess the risk of fracture prior to its occurrence in order to determine whether the participants would have been eligible to intervention to prevent fracture regarding FRAX-intervention thresholds.

### Body composition and sarcopenia assessment

Whole body composition was evaluated by DXA using an Hologic QDR Discovery instrument (Hologic Inc., Waltham, MA, USA). Muscle strength was assessed by isometric HGS measurement, using Jamar^®^ mechanical dynamometer with a precision of 1 kg, either in both hands or in the non-fractured hand. Three repetitive performances were executed with intervals of about 30 s between each run and the maximum value was taken into consideration.

Sarcopenia was determined using the low muscle mass and low muscle strength based on:

1. Baumgartner et al. definition: skeletal mass index (appendicular lean mass relative to height^2^, ALM /height^2^) of ≤ 5.45 kg/m^2^ [35].
2. European working group on sarcopenia in older people (EWGSOP2), without assessment of the chair-stand test (5-times sit-to-stand): skeletal mass index ≤ 5.5 kg/m^2^ and HGS < 16 kg [36].
3. Foundation for the national institute of health (FNIH Sarcopenia project): ratio of appendicular lean mass (ALM) over body mass index (BMI) (ALM/BMI) of < 0.512 and HGS < 16 kg [37].

The severity of sarcopenia, defined by the physical performance tests, such as gait speed, was not estimated. Such tests would be inaccurately low in our population due to the recent fractures, which could contribute to temporary disability.

Obesity was defined as a BMI ≥ 30 kg/m^2^ [38]. Sarcopenic obesity was identified in women who met both the criteria for obesity and low ALM, based on EWGSOP2 criteria [36, 39].

### Bone density and microstructure assessment

Areal BMD (aBMD) was measured at lumbar spine (LS) and hip using the same DXA device used for body composition measurement. According to WHO classification [38], women were categorized as osteoporotic if they had at least one T-score ≤ -2.5 SD (standard deviation) at the lumbar spine, total hip or femoral neck; osteopenic if they had at least one T-score between − 1 and − 2.5 SD with none ≤ -2.5 SD; normal BMD if all T-scores were ≥ -1.0 SD, based on Caucasian women references.

Volumetric BMD (vBMD) and bone microarchitecture variables were measured at distal radius and tibia by HR-pQCT, using an XtremCT instrument (Scanco Medical, Brüttisellen, Switzerland); XtremCT I was used in GERICO and XtremCT II in FLS participants [40]. Data were assessed for all participants using an 80 μm voxel size with internal calibration between two devices to ensure valid and reliable measurements. Due to this internal calibration process, we limited our analysis to the following bone microstructural parameters in tibia and radius that were common to both devices: total, cortical and trabecular area/ total, cortical and trabecular vBMD/ cortical thickness and parameter. The non-dominant limb or the opposite limb in case of fracture was typically examined.

The coefficient of variation (CV) of repeated measurements of BMD by DXA varied between 1.0 and 1.5%. Short-term reproducibility of HRpQCT measurement assessed with repositioning was 0.6-1.0 and 2.8–4.9% for density variables and trabecular microstructure, respectively [41].

### Statistical analysis

Data are reported as medians with interquartile range (iqr) for continuous variables, number and percentage for categorical variables. Characteristics between groups were compared with Kruskal-Wallis or Mann-Whitney test for continuous variables and chi-squared (x^2^) test for categorical variables. Statistical analysis was performed using STATA software, version 14.0. (StataCorp LP, College Station, TX, USA). *P*-values < 0.05 were considered statistically significant.

## Results

### Subjects characteristics

324 women aged over 65 years were identified in the FLS during the screening period and 90 women were included in the study. 45 women meeting the inclusion criteria were identified retrospectively from the GERICO cohort (supplementary Fig. 1).

The median age of participants was 71 (iqr: 13) years, and the median BMI was 25.7 (7.5) kg/m^2^ (Table 1). The most common fracture sites were the hip (26%) followed by the forearm (25%), ankle (22%), humerus (19%), vertebrae (5%) and pelvis (3%) (Table 2). Women with pelvic fractures were excluded from the subgroup analysis by fracture site because of the small sample size (*n* = 3). Women in the hip fracture group were older [median age: 83 [14] years] and had a lower BMI [24 (4.9) kg/m^2^] than women in the ankle fracture group [median age: 70 [6] years old, *p* < 0.001, BMI: 28.7 (7.8) kg/m^2^, *p* = 0.002].

**Table 1:** Population characteristics according to aBMD measured by DXA

aBMD Classification	Total		Osteoporosis		Osteopenia		Νormal		P-value
	***N***	median (iqr) or %	*N*	median (iqr) or %	*N*	median (iqr) or %	*N*	median (iqr) or %	
Age (years)	135	71 (13)	45	75 (11)	72	70 (14)	18	70 (15)	0.272
Weight (kg)	135	67 (18)	45	58 (15)	72	69 (14)^**a**^	18	76 (18)^**a**^	**< 0.001**
Height (cm)	135	160 (9)	45	161 (9)	72	160 (8)	18	161 (7)	0.690
BMI (kg/m^2^)	135	25.7 (7.5)	45	23.7 (5.2)	72	26.1 (5.3)^**a**^	18	30.5 (8.2)^**a**^	**< 0.001**
CCI with age	135	3 (1)	45	3 (2)	72	3 (2)	18	4 (2)	0.793
FRAX score *	128	20 (13)	44	25 (16)	69	19 (10)^**a**^	15	11 (8)^**a**^	**< 0.001**
Tobacco	135	11.9	45	20.0	72	6.9^a^	18	11.1	0.104
Alcohol	135	9.6	45	8.9	72	9.7	18	11.1	0.963
Diabetes (type 2)	135	8.2	45	4.4	72	6.9	18	22.2^**a**^	0.057
Anterior LT fracture	135	41.5	45	53.3	72	37.5	18	27.8	0.107
Morphometric VF grade 2 or 3	126	13.5	43	16.3	67	13.4	16	6.3	0.605
Corticoides use	135	5.2	45	6.7	72	5.6	18	0.0	0.547
VitD/Ca substitution	135	32.6	45	28.9	72	37.5	18	22.2	0.377
Antiresorptive treatment	135	18.5	45	20.0	72	19.4	18	11.1	0.684
Prior or current MHT	135	47.4	45	42.2	72	51.4	18	44.4	0.605
If > 2 falls during last 12months	110	93.6	40	92.5	58	93.1	12	100.0	0.628
***Body composition***									
Total lean mass (g)	130	9816 (2477)	45	8567 (2181)	68	10178 (2153)^**a**^	17	11023 (1592)^**a**^	**< 0.001**
ALM/height^2^ (kg/m^2)^	130	6.2 1.37)	45	5.9 (1.01)	68	6.3 (1.6)^**a**^	17	6.9 (1.2)^**a**^	**< 0.001**
ALM/ BMI	130	0.6 (0.15)	45	0.6 (0.14)	68	0.6 (0.1)	17	0.6 (0.1)	0.129
Total Fat mass (g)	130	6661 (3045)	45	5352 (6262)	68	6975 (2436)^**a**^	17	8065 (2129)^**a**^	**< 0.001**
Total %fat mass	130	40 (9)	45	37 (10)	68	41 (6)^**a**^	17	43 (6)^**a**^	**0.002**
Android % fat mass	130	38 (12)	45	33 (15)	68	39 (10)^**a**^	17	43 (7)^**a**^	**< 0.001**
Gynoid % fat mass	130	42 (7)	45	39 (8)	68	42 (6)^**a**^	17	43 (8)^**a**^	**0.010**
Android/ Gynoid Ratio	130	0.9 (0.21)	45	0.8 (0.3)	68	0.91 (0.2)^**a**^	17	0.9 (0.1)^**a**^	**0.011**
Total abdominal mass (g)	130	2200 (1086)	45	1664 (1155)	68	2370 (857)^**a**^	17	2908 (630)^**a**^	**< 0.001**
Visceral abdominal mass (g)	130	549 (403)	45	412 (343)	68	600 (365)^**a**^	17	637 (334)^**a**^	**< 0.001**
Subcutaneous abdominal mass (g)	130	1643 (864)	45	1312 (824)	68	1725 (599)^**a**^	17	2067 (288)^**a**^	**< 0.001**
***Sarcopenia parameters***									
Low ALM/height^2^, EWGSOP2	130	43.9	45	62.2	68	36.8^a^	17	23.5^**a**^	**0.005**
Low ALM/BMI, FNIH	130	13.1	45	11.1	68	14.7	17	11.8	0.845
Low HGS	127	11.0	43	14.0	66	10.6	18	5.7	0.626
***DXA parameters (SD)***									
Lumbar spine T-score	135	-1.5 (1.8)	45	-2.7(1.4)	74	-1.1 (1.67)^**a**^	16	0.05 (1.3)^**a**^	**< 0.001**
Femoral Neck T-score	132	-1.9 (1.2)	45	-2.7 (0.7)	73	-1.7 (0.8)^**a**^	14	-0.4 (0.8)^**a**^	**< 0.001**
Total hip T-score	132	-1.4 (1.6)	45	-2.3 (0.7)	73	-1.1 (0.9)^**a**^	14	0.1 (0.5)^**a**^	**< 0.001**
One third distal radius T-score	129	-1.5 (1.8)	41	-2.4 (1.7)	72	-1.2 (1.4)^**a**^	16	-0.6 (1.3)^**a**^	**< 0.001**
Ultra distal radius T-score	129	-1.8 (1.9)	41	-2.4 (1.3)	72	-1.6 (1.6)^**a**^	16	-0.7 (1.5)^**a**^	**< 0.001**

aBMD: areal bone mineral density, DXA: bone density scan, BMI: Body mass index, CCI: Charlson Comorbidity Index, MHT: menopausal hormone therapy, LT fracture: low-trauma fracture (major osteroporotic or ankle fracture), ALM: appendicular lean mass, HGS: Hand grip strength, EWGSOP2: European Working Group on Sarcopenia in Older People, FNIH: Foundation for the national institute of health, SD: standard deviation

* With femoral neck BMD inclusion. Actual fracture not included in the algorithm

^a^ p-value < 0.05 versus osteoporosis

Values are median, iqr (interquartile range) or %, p-value < 0.05

**Table 2:** Population characteristics according to fracture site

Fracture Site without pelvis	Hip		Vertebra		Forearm		Humerus		Ankle		P-value
	***N***	median (iqr) or %	***N***	median (iqr) or %	*N*	median (iqr) or %	*N*	median (iqr) or %	*N*	median (iqr) or %	
Age (years)	35	83 (14)	7	70 (12)	34	68 (7)^**a**^	26	72 (9)^**a**^	30	70 (6)^**a**^	**< 0.001**
Weight (kg)	35	60 (15)	7	63 (18)	34	64 (15)	26	70 (18)^**a**^	30	76 (20)^**a**^	**< 0.001**
Height (cm)	35	160 (8)	7	153 (18)	34	162 (7)	26	158 (9)	30	160 (5)	0.395
BMI (kg/m2)	35	24 (4.9)	7	23.5 (8.9)	34	25.6 (4.3)	26	27 (8.9)^**a**^	30	28.7 (7.8)^**a**^	**0.002**
CCI with age	35	4 (3)	7	3 (3)	34	2 (1)^**a**^	26	3 (1)	30	3 (2)^**a**^	**< 0.001**
FRAX score *	32	23 (13)	7	21 (30)	34	15 (16)^**a**^	25	21 (10)	29	15 (10)^**a**^	**0.007**
Tobacco	35	14.3	7	28.6	34	8.8	26	7.7	30	13.3	0.592
Alcohol	35	8.6	7	28.6	34	5.9	26	7.7	30	10	0.442
Diabetes (type 2)	35	8.6	7	0	34	5.9	26	11.5	30	10	0.850
Anterior LT fracture	35	40.0	7	42.9	34	41.2	26	50	30	36.7	0.894
Morphometric VF grade 2 or 3	34	32.4	4	0	33	3.0^**a**^	24	8.3^**a**^	28	0^**a**^	**< 0.001**
Corticoides use	35	5.7	7	28.6	34	0	26	7.7	30	0	**0.011**
VitD/Ca substitution	35	20.0	7	42.9	34	41.2	26	38.5	30	33.3	0.363
Antiresorptive treatment	35	14.3	7	28.6	34	11.8	26	30.1	30	20	0.349
Prioror current MHT	35	34.3	7	57.1	34	52.9	26	46.2	30	56.8	0.387
If > 2 falls during last 12months	32	100.0	7	100	25	84	20	90	23	95.6	0.138
***Body composition***											
Total lean mass (g)	34	9124 (2305)	7	8727 (2032)	34	9515 (1856)	24	10339 (2124)	28	10533 (1969)^**a**^	**0.007**
ALM/height^2^ (kg/m^2)^	34	5.9 (1.3)	7	6.1 (0.7)	34	6.3 (1.1)	24	6.1 (1.8)	28	6.7 (1.2)^**a**^	0.068
ALM/ BMI	34	0.7 (0.2)	7	0.6 (0.2)	34	0.6 (0.1)	24	0.6 (0.2)^**a**^	28	0.6 (0.8)	0.124
Total Fat mass (g)	34	5237 (3112)	7	5999 (2338)	34	6534 (2331)	24	7093 (2903^**a**^	28	8221 (3143)^**a**^	**0.001**
Total %fat mass	34	37 (10)	7	41 (10)	34	41 (5)^**a**^	24	43 (5)^**a**^	28	43 (9)^**a**^	**0.004**
Android % fat mass	34	34 (15)	7	42 (16)^**a**^	34	40 (8)^**a**^	24	40 (9)^**a**^	28	41 (13)^**a**^	**0.003**
Gynoid % fat mass	34	38 (8)	7	41 (8)	34	42 (7)^**a**^	24	42 (7)^**a**^	28	43 (8)^**a**^	**0.026**
Android/ Gynoid Ratio	34	0.8 (0.2)	7	1.0 (0.3)	34	0.9 (0.3)^**a**^	24	0.91 (0.2)	28	0.92 (0.2)^**a**^	0.129
Total abdominal mass (g)	34	1660 (1073)	7	2432 (1381)	34	2236 (664)^**a**^	24	2440 (883)^**a**^	28	2525 (1327)^**a**^	**0.002**
Visceral abdominal mass (g)	34	487 (344)	7	705 (513)	34	500 (473)	24	589 (388)^**a**^	28	666 (527)^**a**^	0.100
Subcutaneous abdominal mass (g)	34	1218 (750)	7	1727 (709)	34	1714 (580)^**a**^	24	1865 (612)^**a**^	28	1889 (840)^**a**^	**< 0.001**
***Sarcopenia parameters***											
Low ALM/height^2^, EWGSOP2	34	61.8	7	42.9	34	41.2	24	50	28	25^**a**^	0.065
Low ALM/BMI, FNIH	34	5.9	7	42.9^**a**^	34	11.8	24	20.8	28	10.7	0.081
Low HGS	34	20.6	7	42.9	32	6.3	25	8	26	0^**a**^	**0.006**
*DXA parameters (SD)*											
Lumbar spine T-score	35	-1.6 (2)	5	-1.7 (0.3)	34	-2.1 (2.02)	25	-1.4 (2.5)	30	-1.1 (1.6)	0.383
Femoral Neck T-score	32	-2.4 (0.8)	7	-1.9 (1.1)	34	-1.7 (1.6)^**a**^	24	-2.0 (1.1)^**a**^	30	-1.4 (1.1)^**a**^	**< 0.001**
Total hip T-score	32	-2.2 (1.1)	7	-1.6 (1.6)	34	-1.2 (1.5)^**a**^	24	-1.1 (1.6)^**a**^	30	-0.7 (1.5)^**a**^	**< 0.001**
One third distal radius T-score	33	-1.9 (1.8)	7	-2.8 (2.1)	30	-1.2 (1.3)^**a**^	26	-1.3 (1.9)	26	-1.1 (1.1)^**a**^	0.068
Ultra distal radius T-score	33	-2.3 (1.2)	7	-1.9 (0.9)	30	-1.8 (1.9)	26	-1.6 (1.9)	26	-1.1 (1.2)^**a**^	0.113

aBMD: areal bone mineral density, DXA: bone density scan, BMI: Body mass index, CCI: Charlson Comorbidity Index, MHT: menopausal hormone therapy, LT fracture: low-trauma fracture (major osteroporotic or ankle fracture), ALM: appendicular lean mass, HGS: hand grip strength, EWGSOP2: European Working Group on Sarcopenia in Older People, FNIH: Foundation for the national institute of health, SD: standard deviation

* With femoral neck BMD inclusion. Actual fracture not included in the algorithm

Values are median, iqr (interquartile range) or %, ^a^ p-value < 0.05 versus hip

Most of participants had osteopenia (54%, *n* = 72), followed by osteoporosis 33% (*n* = 45) and normal BMD (13%, *n* = 18) (Table 1). Osteoporotic women were older and with lower BMI than those with normal BMD. Women with normal aBMD were more likely to be obese (30%, *p* = 0.001) and had a higher prevalence of diabetes mellitus type 2 (22%, *p* = 0.057), compared to women with osteoporosis. The FRAX score was 20% in the total population and 19% in the osteopenic subgroup, both below the intervention threshold of 26% recommended by the Swiss Association Against Osteoporosis for women over 70 years old [42]. In osteoporotic women, the FRAX score was 25%, at the borderline of the intervention threshold (Table 1).

Regarding medication, 33% of the patients received vitamin-calcium supplementation, 19% were treated with anti-osteoporotic medication (bone resorption inhibitors), and 47% were or had been treated with menopausal hormone therapy. There was no difference in the use of these medications according to osteoporosis status.

In terms of body composition, fat and lean mass parameters were lower in osteoporotic women compared to women with osteopenia or normal BMD, and in those with hip fractures compared to other fracture sites (*p* < 0.05, for most variables but not for ALM/BMI) (Tables 1 and 2). Furthermore, bone microstructure analysis showed that vBMD values at the radius and tibia were significantly lower (*p* < 0.05) in osteoporotic women compared to women with normal BMD, and in those with hip fractures compared to ankle fractures. Parameters such as area, thickness, and perimeter were generally higher in women with normal BMD and those with ankle fractures compared to women with osteoporosis and hip fractures, respectively, although not all differences reached statistical significance (supplementary Tables 1 and 2).

### Prevalence of sarcopenia and obesity

**Fig. 1:**
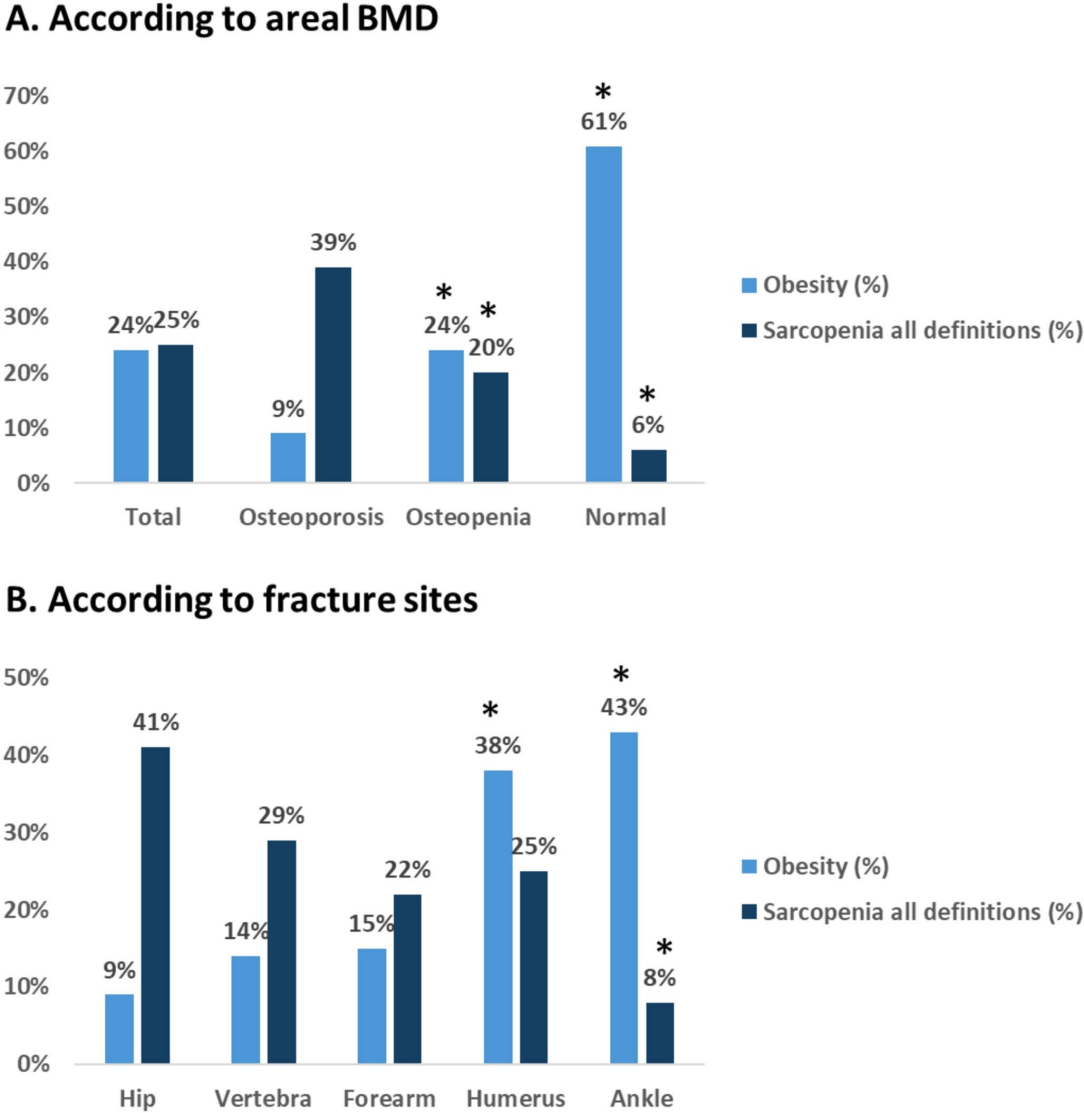
Prevalence of obesity and sarcopenia according to osteoporotic status (**A**) or fracture sites (**B**). * *p* < 0.05 compared to osteoporosis (**A**) or hip fracture (**B**) groups

The overall prevalence of sarcopenia in the study population, regardless of the definition used, was 25% (Fig. 1A**).** Sarcopenia was mostly prevalent among osteoporotic women (39%, *p* = 0.014), those with hip fractures (41%, *p* = 0.069) and vertebral fractures (29%, *p* > 0.05) compared to women with normal BMD and ankle fractures. In contrast, only 6% of women with normal BMD and 8% of those with ankle fractures were sarcopenic (*p* < 0.05, compared to women with osteoporosis and hip fractures) (Fig. 1B).

**Fig. 2:**
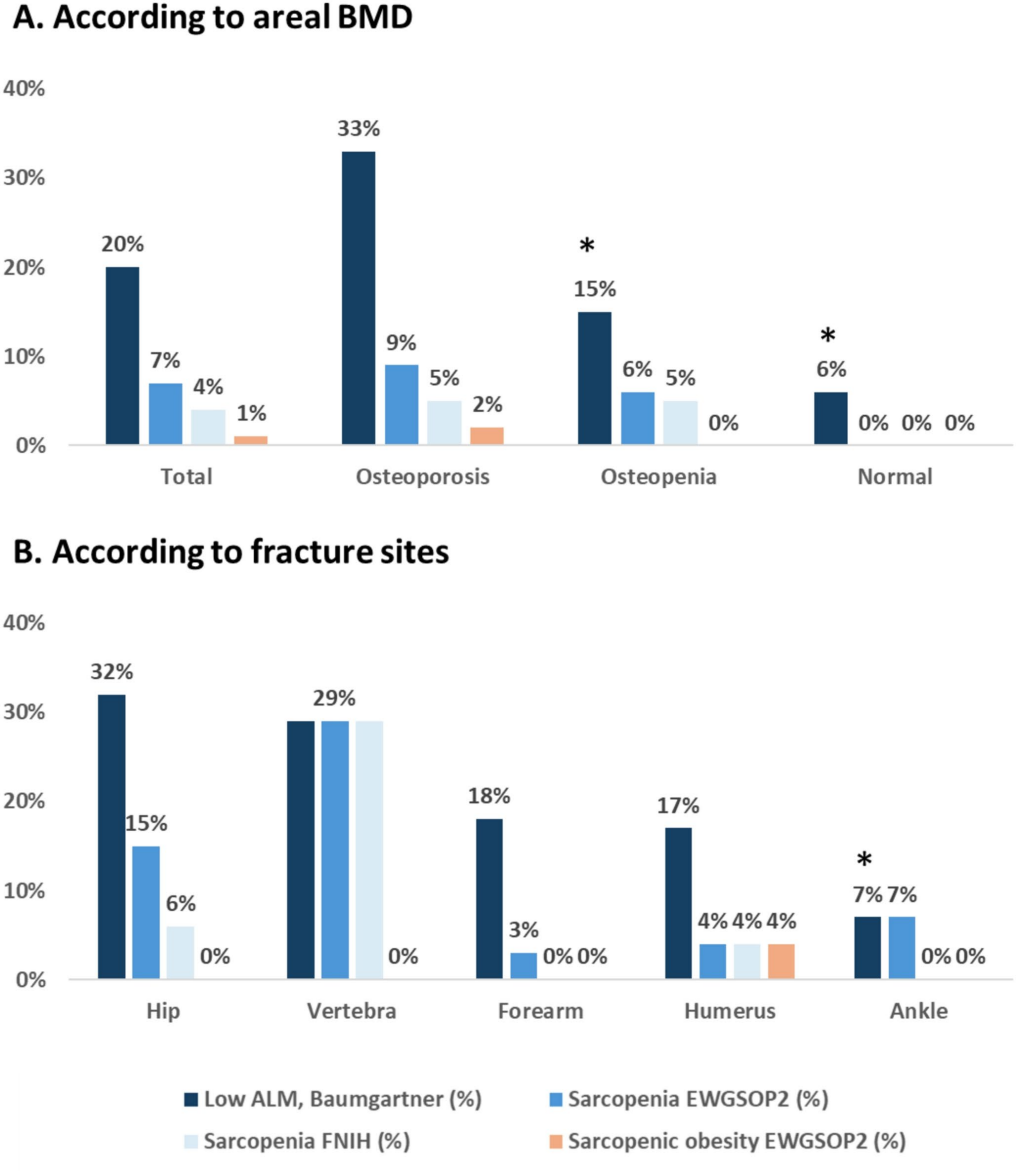
Prevalence of sarcopenia using the various definitions according to osteoporotic status (**A**) or fracture sites (**B**). * *p* < 0.05 compared to osteoporosis (**A**) or hip fracture (**B**) groups

More women were classified as sarcopenic in total population (20%) and subgroups (33% in osteoporotic, 15% in osteopenic, 6% in women with normal BMD) (Fig. 2A) using Baumgartner’s criteria compared to the adapted EWGSOP2 and FNIH definitions. However, due to the adapted EWGSOP2 criteria and the lack of physical performance measurements, the prevalence of sarcopenia may be underestimated (Fig. 2A and B).

The overall prevalence of obesity was 24% (*p* = 0.040). Obesity was most prevalent in normal BMD group (61% versus 9% in osteoporotic women, *p* < 0.05) and among women with ankle and humerus fractures (43% and 38%, respectively versus 9% in women with hip fracture, *p* < 0.05) (Fig. 1A and B). Only two women with sarcopenic obesity were identified, both had osteoporosis and humerus fractures. Overall, 33% of women with normal BMD and 56% of osteopenic women were neither obese nor sarcopenic. When analyzed by fracture site, the proportion of women without body composition alterations (i.e., neither obese nor sarcopenic) was 50% for hip fractures (41% were sarcopenic and 9% obese), 57% for vertebral fractures (29% sarcopenic and 14% obese), 63% for forearm fractures (22% sarcopenic and 15% obese), 37% for humeral fractures (25% sarcopenic and 38% obese), and 49% for ankle fractures (8% sarcopenic and 43% obese) (Fig. 1B).

### Contribution of bone microstructure analysis in the mechanism of fracture in non-osteoporotic women

**Fig. 3:**
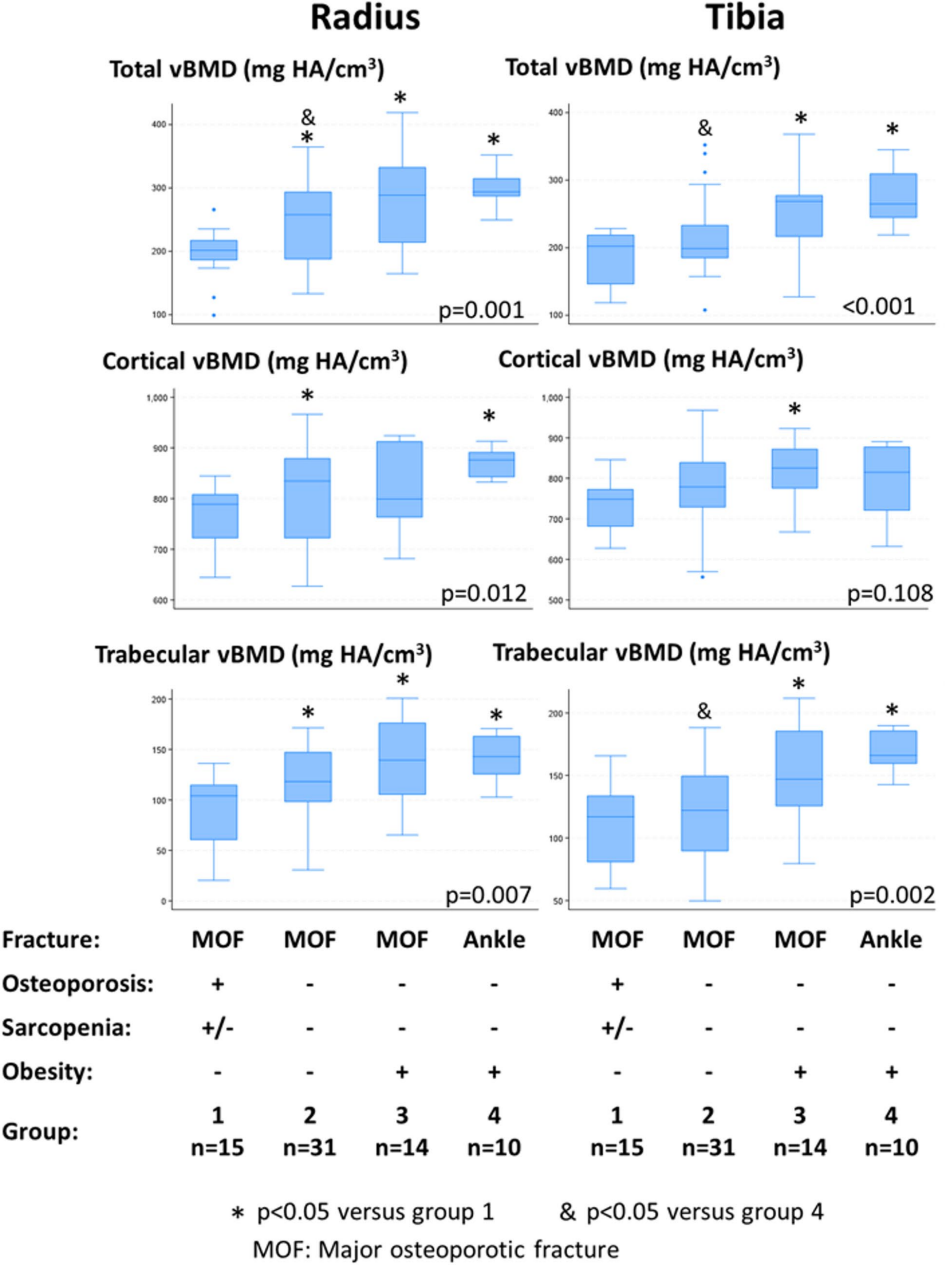
Volumetric bone mineral density (vBMD) at the distal radius and tibia according to fracture site, obesity, osteoporotic ± sarcopenic status

Last we explored whether bone microstructural alterations may be determinants of the occurrence of major osteoporotic fractures in women without osteoporosis nor sarcopenia, in two groups stratified for obesity (group 2, *n* = 31 and group 3, *n* = 14, respectively). We compared their bone microstructural characteristics to two controls groups with “extreme phenotype”: a “most fragile” bone group of non-obese women with MOF and osteoporosis (group 1); a “least fragile” bone group of obese non-osteoporotic/non-sarcopenic women with ankle fracture, which is not a MOF (group 4) (Fig. 3). Women with MOF but without osteoporosis, sarcopenia nor obesity (group 2) had slightly higher total vBMD at the radius and tibia compared to the “most fragile” bone group (group 1), but the differences between these two groups were not significant for cortical and trabecular vBMD at the tibia (Fig. 3), cortical thickness at the tibia, and trabecular spacing at the radius and tibia (data not shown). These data indicate that these two groups share some microarchitectural characteristics of bone fragility which may have contributed to the occurrence of MOF despite a non-osteoporotic status. Conversely, obese women with MOF but without osteoporosis nor sarcopenia (group 3) exhibited microstructural similarities with the “least fragile” bone group (group 4), composed of obese women with ankle fractures. There were indeed no significant differences between groups 3 and 4 in total, cortical and trabecular vBMD (Fig. 3), cortical area and thickness, and trabecular spacing at the radius and tibia (data not shown). These data suggest that MOF in obese women may occur in the absence of bone microstructural defect nor sarcopenia, similarly as ankle fractures.

## Discussion

In this observational, descriptive study, we estimated the prevalence of sarcopenia and obesity in postmenopausal women who had recently sustained low-trauma fractures. Additionally, we aimed to characterize bone microstructural alterations at the time of fracture. Our objective was to gain further insight into the mechanisms underlying fractures in community-dwelling women.

The overall prevalence of sarcopenia, across all definitions, was 25%, lower than expected when compared to other studies [20, 43–45]. Sarcopenia prevalence varies depending on the population studied [46]. In our study, we intentionally excluded women with severe comorbidities and cognitive impairment, which likely led to an underestimation of sarcopenia prevalence. However, this was consistent with our aim to study a community-dwelling population. Sarcopenia was identified in only 6% of women with normal BMD and 8% of those with ankle fractures. Similarly, in a cohort of 5,911 community-dwelling individuals, the prevalence of sarcopenia was also low (4.4%) [47]. In a systematic review, the prevalence of sarcopenia ranged from 0.7 to 30.1% in community-dwelling women, aged ≥ 50 years old [46]. These findings suggest that sarcopenia may not represent a major contributor of fracture in this population.

However, sarcopenia was common among women with osteoporosis (39%) and those with hip fractures (41%) supporting the concept of ‘osteo-sarcopenia’ [48]. Consistent with other studies, the coexistence of sarcopenia and low BMD or low BMD alone appears to be associated with increased fracture risk, whereas sarcopenia alone does not [18, 19, 47, 48]. These observations imply that women with osteoporosis and/or a history of hip or vertebral fractures should be systematically screened for sarcopenia and may benefit from targeted fall prevention programs and interventions promoting muscle health [49].

In our study, we used adapted definitions of sarcopenia based on three common established definitions. The earliest criteria, proposed by Baumgartner, classified a higher proportion of women as sarcopenic (20%) compared to more recent definitions. Additionally, the prevalence of sarcopenia was higher when using the adapted EWGSOP2 (7%) than the FNIH definition (4%) likely due to differences in diagnostic criteria; EWGSOP2 uses height-adjusted indices, whereas FNIH relies on BMI-adjusted thresholds. However, our results tend to underestimate the prevalence of sarcopenia first because the chair-stand test was not included in the application of the EWGSOP2 criteria and secondly because we did not assess sarcopenia severity through physical performance measures. Nevertheless, previous studies have suggested that EWGSOP2 criteria are more sensitive than FNIH criteria for the detection of sarcopenia [48, 50] and are also more effective in predicting fall risk [51, 52]. Although all diagnostic criteria for sarcopenia have been associated with incident osteoporotic fractures, the most appropriate definition for use in community-dwelling populations remains unclear [53].

The overall prevalence of obesity in our study population was 24%, with particularly high rates among women with normal BMD and those with ankle or humerus fractures (61%, 43%, and 38%, respectively), similar with previous findings [25, 27, 54, 55]. Obesity has been associated with a 30% increased risk of proximal humerus fractures [24] and a significantly higher incidence of ankle fractures compared to non-obese women [25]. Ankle and humerus fractures are more commonly observed in obese women than classic osteoporotic fractures [28, 54, 56, 57], suggesting that obesity may represent a specific risk factor for these fracture types because excess body weight may exert greater mechanical stress on bones during falls [58]. Moreover, bone microstructure analysis indicated that obese individuals exhibited higher vBMD and thicker cortical bones compared to non-obese individuals. Increased body weight is likely associated with greater bone mass, a result that does not explain the occurrence of fractures in this population [55, 59].

In our study, we identified 24 obese individuals without osteoporosis or sarcopenia, presenting a MOF or ankle fracture (groups 3 and 4, respectively). The bone microstructure analysis indicated that these two groups exhibited similar microstructural characteristics across most variables, despite differing fracture types. Their microarchitectural characteristics were however less altered than non-obese women with MOF (groups 1 and 2). These data suggest that some low-trauma fractures occurring at bone sites of MOF may not be fragility fractures, in the absence of any criteria of fragility regarding the bone and muscular phenotype. Conversely, previous analyses in our group and others reported that some microarchitectural bone defects may also be identified in women with ankle fractures [41]. The mechanism of injury, rather than bone fragility, may also play a significant role in fracture occurrence in some individuals [24, 25, 27, 56, 60]. Furthermore, 31 women (23%), non-osteoporotic, non-sarcopenic and with normal BMI were identified (group 2) who, surprisingly, did not differ significantly from the 15 osteoporotic and/or sarcopenic women with MOF (group 1) in certain bone microstructural parameters (cortical and trabecular vBMD in the tibia, cortical thickness in the tibia, or trabecular spacing in both the radius and tibia). This similarity may be attributed to the osteoporotic nature of their fractures, despite normal BMD values.

These results suggest that bone microstructure analysis may potentially serve as a valuable tool for fracture risk assessment, especially in women with normal BMD. aBMD can be falsely elevated as DXA tends to overestimate BMD measurements in the presence of artifacts, such as osteophytes and lumbar lesions [61, 62]. Moreover, aBMD is typically higher in obese compared to non-obese women and that may underestimate the increased fracture risk associated with obesity [63]. In such cases, aBMD measurements at peripheral bone sites, such as ultra-distal radius and the assessment of peripheral cortical and trabecular vBMD and microstructure, may provide a more accurate prediction of fracture risk than DXA alone [29]. However, there is a lack of well-established microstructural fragility thresholds [10, 64].

This study was conducted in community-dwelling, post-menopausal women with recent fragility fracture. Our purpose was to comprehensively elucidate the various determinants of fracture investigating both the body composition and bone microstructural characteristics and examine whether these characteristics could be incriminated in the occurrence of LT fractures in women without osteoporosis. For this reason, the most frequent fractures were taken into account, including MOF but also ankle fractures to provide a broader perspective with fracture sites not classically associated with osteoporosis. Based on these data, we believe that secondary fracture prevention strategies should be more specifically tailored according to body composition status. Management of obesity and metabolic syndrome, when present, is warranted given the high prevalence of obesity in these women with fracture. Nutritional interventions focusing on adequate protein intake, along with muscle-strengthening strategies, would be especially appropriate for patients with a predominantly sarcopenic profile. In patients with normal BMD and no evidence of bone microarchitectural impairment, the benefit of anti-osteoporotic treatment appears questionable, even in the presence of a fracture likely driven by extra-skeletal factors.

This study has, however, some limitations. The study is descriptive in nature, without longitudinal follow-up. Despite an initially large screening of patients with fractures, the final number of women meeting the inclusion criteria or accepting to participate was relatively small, particularly after subdivision into phenotypic or fracture site subgroups which reduces the statistical power and may limit the generalizability of our findings. Additionally, the exclusion of women with severe comorbidities and/or cognitive impairment as well as the lack of strength parameter measurement (chair-stand test) may have led to an important underestimation of the prevalence of sarcopenia. But our aim was to focus on community-dwelling, postmenopausal women with recent fragility fractures and without severe comorbidities which may have overestimated the prevalence of osteoporosis and sarcopenia. Finally, the severity of sarcopenia, assessed through physical performance, was not evaluated due to the difficulty of performing gait speed tests in patients with recent fractures.

In conclusion, a substantial proportion of women with apparent low-trauma fracture, including at sites of called MOF, do not have osteoporosis nor alterations of body composition, i.e. sarcopenia or obesity. These data suggest that some non-traumatic fractures may not necessarily qualify as fragility fractures, in the absence of identification of bone fragility in terms of bone mass or microstructure, poor muscle condition or obesity. The mechanisms underlying fractures in non-osteoporotic women with normal body composition remain unclear, and further research is needed to explore this area. Beyond the level of trauma, the spatial configuration of the fall or trauma applied to a specific bone geometry, beyond the intensity of the strength applied on the bone, may lead to fractures in the absence of underlying fragility. Secondary fracture prevention in these patients with MOF without alterations of body composition should prioritize fall prevention strategies, the maintenance of adequate nutritional intake, and the promotion of weight-bearing physical activity. In women without osteoporosis but experiencing fracture, obesity is much more prevalent than sarcopenia. In women with osteoporosis, sarcopenia is more frequent. Therefore, FLS programs should incorporate strategies that address both ‘osteo-sarcopenia’ and obesity, in addition to osteoporosis management and fall risk prevention.

## Supplementary Information

Below is the link to the electronic supplementary material.

Supplementary Material 1

Supplementary Material 2

## Data Availability

No datasets were generated or analysed during the current study.
