# Supplementary figures and images for: Are low-trauma fractures all fragility fractures? Insights into musculoskeletal and body composition characteristics of community-dwelling post-menopausal women with a recent fracture
Source: Aging Clin Exp Res. 2025 Aug 13;37(1):245. doi: 10.1007/s40520-025-03154-w (PMC12350515; doi:10.1007/s40520-025-03154-w)

**Supplementary Figure 1: Flowchart of study population**


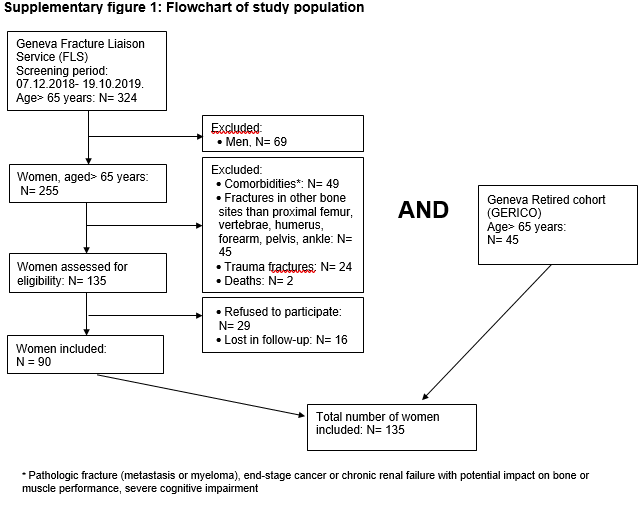

Supplement: Supplementary file 1 — Supplementary Material 1 [file 40520_2025_3154_MOESM1_ESM.docx]
