# Supplementary material for: Are low-trauma fractures all fragility fractures? Insights into musculoskeletal and body composition characteristics of community-dwelling post-menopausal women with a recent fracture
Source: Aging Clin Exp Res. 2025 Aug 13;37(1):245. doi: 10.1007/s40520-025-03154-w (PMC12350515; doi:10.1007/s40520-025-03154-w)
Supplement: Supplementary file 2 — Supplementary Material 2 [file 40520_2025_3154_MOESM2_ESM.docx]

| **Supplementary table 1: Bone variables measured by HR-pQCT, classified according to aBMD** | | | | | | | | | |
| --- | --- | --- | --- | --- | --- | --- | --- | --- | --- |
| **aBMD Classification** | **Total** | | **Osteoporosis** | | **Osteopenia** | | **Νormal** | | **P-value** |
|  | **N** | **median (iqr)** | **N** | **median (iqr)** | **N** | **median (iqr)** | **N** | **median (iqr)** |  |
| ***in distal radius*** | | | | | | | | | |
| Total Area (mm^2^) | 123 | 269.7 (51.6) | 38 | 278.3 (49.8) | 68 | 258.5 (51.7) | 17 | 282.9 (13.8) | **0.039** |
| Cortical Area (mm^2^) | 119 | 35.9 (17.7) | 36 | 29.1 (11.3) | 66 | 38.85 (17.8)^a^ | 17 | 42.1 (14.9)^a^ | **<0.001** |
| Trabecular Area (mm^2^) | 119 | 221.2 (52) | 36 | 234.7 (48.1) | 66 | 210.25 (55)^a^ | 17 | 228.3 (30.7) | **0.024** |
| Total vBMD (mg HA/cm^3^) | 119 | 248.2 (105.8) | 36 | 187.4 (75.5) | 66 | 265.1 (85.7)^a^ | 17 | 279.9 (79.9)^a^ | **<0.001** |
| Cortical vBMD (mg HA/cm^3^) | 119 | 812.6 (126) | 36 | 754.6 (107) | 66 | 832.75 (114.2)^a^ | 17 | 834.2 (120.9)^a^ | **0.001** |
| Cortical thickness (mm) | 119 | 0.5 (0.3) | 36 | 0.4 (0.1) | 66 | 0.6 (0.3)^a^ | 17 | 0.6 (0.2)^a^ | **<0.001** |
| Cortical perimeter (mm) | 119 | 69.6 (7.3) | 36 | 70.5 (6.2) | 66 | 68.8 (8.1) | 17 | 72.4 (3.9) | **0.040** |
| Trabecular vBMD (mg HA/cm3) | 119 | 116.3 (51.5) | 36 | 92.2 (60.9) | 66 | 119.6 (37.5)^a^ | 17 | 136 (57.9)^a^ | **<0.001** |
| ***in tibia*** | | | | | | | | | |
| Total Area (mm^2^) | 129 | 701.2 (152.2) | 41 | 707.9 (147.9) | 71 | 695.8 (139.7) | 17 | 725.4 (90.4) | 0.415 |
| Cortical Area (mm^2^) | 127 | 77.6 (36.7) | 40 | 61.85 (25.75) | 70 | 83.7 (26.8)^a^ | 17 | 100.5 (30.5)^a^ | **<0.001** |
| Trabecular Area (mm^2^) | 127 | 600 (174.2) | 40 | 618.8 (171.9) | 70 | 581.5 (156) | 17 | 593 (124.4) | 0.194 |
| Total vBMD (mg HA/cm^3^) | 127 | 216.1 (73) | 40 | 187.9 (66.9) | 70 | 229 (80.7)^a^ | 17 | 268.3 (95.5)^a^ | **<0.001** |
| Cortical vBMD (mg HA/cm^3^) | 127 | 773.6 (119.2) | 40 | 737.1 (93.4) | 70 | 795.5 (87.9)^a^ | 17 | 810.2 (119.9)^a^ | **0.001** |
| Cortical thickness (mm) | 127 | 0.75 (0.4) | 40 | 0.6 (0.3) | 70 | 0.8 (0.3)^a^ | 17 | 0.88 (0.4)^a^ | **<0.001** |
| Cortical perimeter (mm) | 127 | 103.2 (12) | 40 | 102.85 (12.5) | 70 | 102.5 (11.6) | 17 | 103.3 (6.6) | 0.704 |
| Trabecular vBMD (mg HA/cm3) | 127 | 129.8 (54.9) | 40 | 108.8 (44.7) | 70 | 134.9 (50.6)^a^ | 17 | 150.9 (59.5)^a^ | **<0.001** |
| Iqr: interquartile range, HR-pQCT: high-resolution peripheral quantitative computerized tomography, vBMD volumetric bone mineral density, SD: Standard deviation | | | | | | | | | |
| ^a^ p-value< 0.05 versus osteoporosis | | | | | | | | | |

| **Supplementary table 2: Bone variables measured by HR-pQCT, classified according to fracture site** | | | | | | | | | | | |  |
| --- | --- | --- | --- | --- | --- | --- | --- | --- | --- | --- | --- | --- |
| **Fracture Site without pelvis** | **Hip** | | **Vertebra** | | **Forearm** | | **Humerus** | | **Ankle** | | **P-value** |  |
|  | **N** | **median (iqr)** | **N** | **median (iqr)** | **N** | **median (iqr)** | **N** | **median (iqr)** | **N** | **median (iqr)** |  |  |
| ***in distal radius*** | | | | | | | | | | | |  |
| Total Area (mm^2^) | 32 | 279.1 (45.9) | 6 | 283.8 (67.7) | 29 | 277.1 (36.4) | 23 | 266.3 (90.7) | 29 | 262.5 (55.9)^a^ | 0.428 |  |
| Cortical Area (mm^2^) | 32 | 31.1 (13.6) | 6 | 33.3 (26.1) | 28 | 38.4 (22.8)^a^ | 22 | 36.3 (19.3) | 27 | 38.7 (10.4)^a^ | **0.03** |  |
| Trabecular Area (mm^2^) | 32 | 234.1 (42.1) | 6 | 238.1 (89.4) | 28 | 218 (46.7) | 22 | 213.6 (59.7) | 27 | 209.7 (51.5)^a^ | 0.244 |  |
| Total vBMD (mg HA/cm^3^) | 32 | 194.8 (92.0) | 6 | 215.6 (146.9) | 28 | 251.4 (89.9)^a^ | 22 | 244.8 (131) | 27 | 274 (51.5)^a^ | **0.028** |  |
| Cortical vBMD (mg HA/cm^3^) | 32 | 766.1 (145.4) | 6 | 771.2 (163.7) | 28 | 836.3 (140.5)^a^ | 22 | 816.3 (114.3) | 27 | 834.2 (81.7)^a^ | **0.021** |  |
| Cortical thickness (mm) | 32 | 0.4 (0.2) | 6 | 0.47 (0.5) | 28 | 0.6 (0.4)^a^ | 22 | 0.5 (0.3) | 27 | 0.6 (0.2)^a^ | **0.014** |  |
| Cortical perimeter (mm) | 32 | 70.7 (5.3) | 6 | 71.4 (9.6) | 28 | 69.3 (7.5) | 22 | 69 (9.1) | 27 | 69.1 (8.3)^a^ | 0.278 |  |
| Trabecular vBMD (mg HA/cm^3^) | 32 | 102.2 (58.2) | 6 | 116.65 (50) | 28 | 116.3 (45.2) | 22 | 116.8 (76.8) | 27 | 128.4 (45)^a^ | 0.066 |  |
| ***in tibia*** | | | | | | | | | | | |  |
| Total Area (mm^2^) | 31 | 733.3 (181.5) | 7 | 664 (227.8) | 34 | 704.9 (144.3)^a^ | 25 | 684.4 (171.9) | 27 | 700.3 (119.4)^a^ | 0.178 |  |
| Cortical Area (mm^2^) | 31 | 54.3 (33.5) | 7 | 83.6 (47.8) | 34 | 83.1 (24.7)^a^ | 24 | 84.2 (33.7)^a^ | 26 | 82 (26.4)^a^ | **<0.001** |  |
| Trabecular Area (mm^2^) | 31 | 675.7 (167.9) | 7 | 591 (269.8) | 34 | 602.1 (136.6)^a^ | 24 | 566 (204.7)^a^ | 26 | 586.1 (123)^a^ | **0.03** |  |
| Total vBMD (mg HA/cm^3^) | 31 | 173 (67.7) | 7 | 184.1 (85.1) | 34 | 222.7 (52.6)^a^ | 24 | 212.3 (86.4)^a^ | 26 | 247.9 (67.1)^a^ | **<0.001** |  |
| Cortical vBMD (mg HA/cm^3^) | 31 | 701.9 (133.4) | 7 | 778.9 (125.8) | 34 | 794.2 (76.2)^a^ | 24 | 801.5 (98.4)^a^ | 26 | 793.2 (100.4)^a^ | **<0.001** |  |
| Cortical thickness (mm) | 31 | 0.5 (0.33) | 7 | 0.75 (0.61) | 34 | 0.82 (0.24)^a^ | 24 | 0.865 (0.4)^a^ | 26 | 0.8 (0.3)^a^ | **<0.001** |  |
| Cortical perimeter (mm) | 31 | 106.3 (12.6) | 7 | 100.4 (17.6) | 34 | 103.4 (10.9) | 24 | 101 (14.3) | 26 | 102.3 (10) | 0.25 |  |
| Trabecular vBMD (mg HA/cm3) | 31 | 122.1 (54.8) | 7 | 121.7 (27.5) | 34 | 123.8 (47.5) | 24 | 131.3 (44.3) | 26 | 149.3 (72.2)^a^ | 0.088 |  |
| Iqr: interquartile range, HR-pQCT: high-resolution peripheral quantitative computerized tomography, vBMD volumetric bone mineral density, SD: Standard deviation | | | | | | | | | | | |  |
| ^a^ p-value< 0.05 versus hip | | | | | | | | | | | |  |
